# The Square-Plus Flap: A Modification to Release Long Postburn Scar Contractures

**DOI:** 10.1055/a-2189-9767

**Published:** 2024-02-07

**Authors:** Mahmoud A. Hifny, Rei Ogawa

**Affiliations:** 1 Department of Plastic Surgery, Faculty of Medicine, Qena University Hospital, South Valley University, Qena, Egypt; 2 Department of Plastic, Reconstructive and Aesthetic Surgery, Nippon Medical School, Tokyo, Japan

**Keywords:** square flap, Z-plasty, burn, contractures, scar

## Abstract

The square flap method has been successful in releasing contracture bands at various body regions. However, the original square flap method alone may not be efficient in releasing long contracture bands. We, therefore, proposed an extended design to the traditional design, which is called the “square-plus flap.” A 4-year-old girl presented with a postburn web-like contracture band over the right axilla. We marked a square flap technique at the center of the contracture band and then two additional Z-plasties were placed on both edges of the flap. After the release and securing of the square flap, the adjacent distal Z-plasty was then transposed and sutured in their new locations. We do not need to incise the proximal Z-plasty as we could achieve complete relaxation of the contracture band. This novel modification can be added to the plastic surgeon's armamentarium for releasing long postburn contracture bands involving distinct body regions.

## Introduction

The square flap method was originally described by Hyakusoku in 1987. It is considered a type of local flap that combines transposition and advancement technique that is appropriate for the correction of a single, linear contracture band. 1 The square flap method has been successfully used as an effective way to release band contractures at various locations involving axilla, elbow, and digital web spaces. 2 3 However, the original square flap method alone may not be efficient enough to release the whole band in cases of long-scar contracture bands. We, therefore, proposed an extended design to the traditional square flap method, which is called “square-plus flap.”

## Idea

A 4-year-old girl presented to our clinic with a postburn web-like axillary contracture band involving the right anterior axillary fold. The preoperative contracture band length was 11 cm and the degree of abduction was 120 degrees. We found that an application of a classic square flap method alone is not adequate to release a relatively long, linear contracture band. We, therefore, decided to extend the design of the traditional square flap.

The contracture band was divided into three equal parts, the central part was used for the square flap method and the two lateral parts were used for Z-plasties. Along the center of the contracture band, we marked a square on one side of the contracture and two triangular flaps on the other side of the contracture. The angle of the first triangular flap and the second flap were kept at 45 and 90 degrees, respectively, while the angles of the two Z-plasties were 45 degrees. The lengths of the square sides, triangular flaps, and Z-plasty limbs were kept equal.

**Fig. 1:**
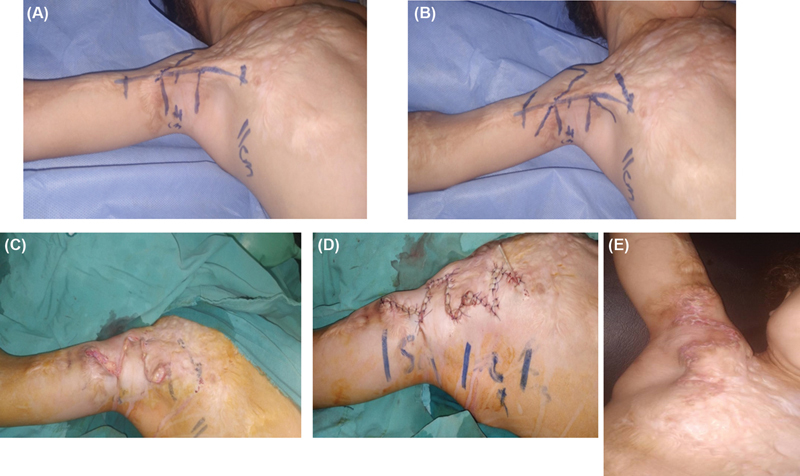
( **A** ) Long contracture band over right anterior axillary line and square flap design. ( **B** ) Addition of double opposing Z-plasty on either side of the square flap. ( **C** ) After the square flap was advanced and the adjoining triangular flaps were positioned on each side of the square flap. ( **D** ) Adjacent distal Z-plasty was then incised, transposed, and sutured in their new locations. ( **E** ) A follow-up picture at 6 weeks postoperatively.

A full-thickness skin incision was made at first along the marked square flap design, followed by an incision in the subcutaneous tissue, and all contracted scar tissue was released. After release, the square flap was advanced across the contracture area, and the adjoining triangular flaps were transposed and then placed proximally and distally on each side of the square advancement flap. Following securing the square flap, we assessed the efficacy of elongation of the contracture band and thereafter decided to proceed with the addition of Z-plasty in order to release any residual contracture. The adjacent distal Z-plasty was then incised, transposed, and sutured in their new locations ( Fig. 1 ). In our illustrated clinical case, we did not need to incise the proximal Z-plasty as we could effectively achieve complete relaxation and elongation of the contracture band. The postoperative contracture band length was 14 cm when we used the square flap method alone and the length was increased to 15.5 cm with the addition of distal Z-plasty. Also, the degree of abduction was improved from 120 degrees preoperatively to 180 degrees postoperatively.

## Discussion

We developed a new modification of the square flap method, combining a single square flap plus one or two opposing Z-plasties. This modification is generally applicable to release long postburn scar contractures. The standard square flap method is a type of local flap that is appropriate for the surgical release of a single, linear band contracture at various locations that have adjacent healthy tissue. 2 Classically, it consists of a square advancement flap incorporated with two triangular transposition flaps.

The square flap method has been demonstrated to be suitable for Kurtzman type IIa and IIb axillary web scar contractures. 2 4 5 Also, it possesses numerous advantages, particularly in web-like contracture release involving the axilla, it adequately restores the original web architecture, provides the largest vascularized flap area with the least physiological tension, and delivers better lengthening when compared with other Z-plasties. 6

**Fig. 2:**
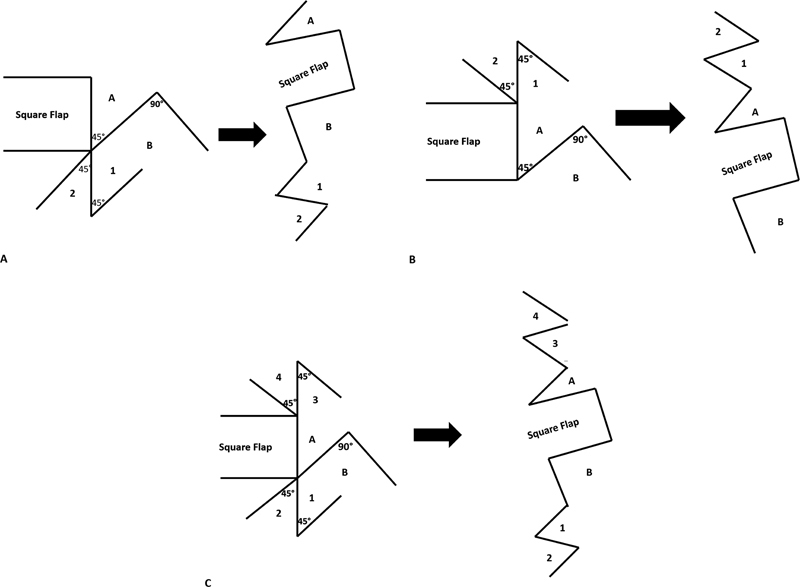
( **A** ) Square-plus flap type IA: addition of one Z-plasty proximal to the designed square flap. ( **B** ) Square-plus flap type IB: addition of one Z-plasty distal to the designed square flap. ( **C** ) Square-plus flap type II: addition of double opposing Z-plasty on either side of the square flap.

However, when used to release a long contracture band, utilization of a single large flap is susceptible to excessive transverse tension which may be associated with wound dehiscence or ischemic tissue necrosis. To avoid these problems, we introduce a square plus modification which includes the addition of two Z-plasties on both sides to the classically designed square flap. In our technique, following insetting of the traditional square flap, we assessed the efficacy of elongation of the band provided by the square flap alone and we then decided to proceed with using either single or double Z-plasties in order to overcome any residual contracture and provide more length gain. In our illustrated clinical case, we could effectively achieve complete relaxation and elongation of the contracture band from 11 cm preoperatively to 14 cm postoperatively when we used the square flap method alone. However, the gain in length of the contracture band was further increased to 15.5 cm with the addition of distal Z-plasty. This indicates further improvement in the length gain of the contracture band with the addition of Z-plasties to the traditional square flap method. We classified the modified square plus method into type IA which involves the addition of one Z-plasty proximal to the designed square flap, type IB involves the addition of one Z-plasty distal to the designed square flap, and type II which includes the addition of double opposing Z-plasty on either side of the designed square flap ( Fig. 2 ; Table 1 ). The application of square plus flap in this consequently is effective in releasing long postburn scar contractures that provide suitable lengthening with lesser transverse tension.

**Table 1:** Classification of square plus flap

Square-plus flap types	
Type IA	Addition of one Z-plasty proximal to the designed square flap
Type IB	Addition of one Z-plasty distal to the designed square flap
Type II	Addition of double opposing Z-plasty on either side of the square flap

We believe that our modification is a more reliable and versatile method than commonly described Z-plasty techniques, such as Z-plasties in series, seven- and nine-flap Z-plasties, in long-scar contracture release, which gives good lengthening with lesser transverse tension. By using our proposed technique, we preserve the advantage of the square flap which accurately reproduces the original axillary web architecture with better lengthening and avoids tip necrosis that is associated with multiple Z-plasty techniques, especially in scarred skin. However, long-term follow-up is needed in order to confirm the efficiency of this modification.

This novel modification can be added to the plastic surgeon's armamentarium for releasing long postburn contracture bands involving distinct body regions.
